# Serotonergic system antagonists target breast tumor initiating cells and synergize with chemotherapy to shrink human breast tumor xenografts

**DOI:** 10.18632/oncotarget.16646

**Published:** 2017-03-29

**Authors:** William D Gwynne, Robin M Hallett, Adele Girgis-Gabardo, Bojana Bojovic, Anna Dvorkin-Gheva, Craig Aarts, Kay Dias, Anita Bane, John A Hassell

**Affiliations:** ^1^ Department of Biochemistry and Biomedical Sciences, McMaster University, Canada; ^2^ Department of Pathology and Molecular Medicine, McMaster University, Canada

**Keywords:** breast cancer stem cells, tumor-initiating cells, serotonin antagonists, antidepressants, cytotoxic chemotherapy

## Abstract

Breast tumors comprise an infrequent tumor cell population, termed breast tumor initiating cells (BTIC), which sustain tumor growth, seed metastases and resist cytotoxic therapies. Hence therapies are needed to target BTIC to provide more durable breast cancer remissions than are currently achieved. We previously reported that serotonergic system antagonists abrogated the activity of mouse BTIC resident in the mammary tumors of a HER2-overexpressing model of breast cancer. Here we report that antagonists of serotonin (5-hydroxytryptamine; 5-HT) biosynthesis and activity, including US Federal Food and Drug Administration (FDA)-approved antidepressants, targeted BTIC resident in numerous breast tumor cell lines regardless of their clinical or molecular subtype. Notably, inhibitors of tryptophan hydroxylase 1 (TPH1), required for 5-HT biosynthesis in select non-neuronal cells, the serotonin reuptake transporter (SERT) and several 5-HT receptors compromised BTIC activity as assessed by functional sphere-forming assays. Consistent with these findings, human breast tumor cells express TPH1, 5-HT and SERT independent of their molecular or clinical subtype. Exposure of breast tumor cells *ex vivo* to sertraline (Zoloft), a selective serotonin reuptake inhibitor (SSRI), reduced BTIC frequency as determined by transplanting drug-treated tumor cells into immune-compromised mice. Moreover, another SSRI (vilazodone; Viibryd) synergized with chemotherapy to shrink breast tumor xenografts in immune-compromised mice by inhibiting tumor cell proliferation and inducing their apoptosis. Collectively our data suggest that antidepressants in combination with cytotoxic anticancer therapies may be an appropriate treatment regimen for testing in clinical trials.

## INTRODUCTION

Breast cancer was the first malignancy of epithelial tumors reported to follow the cancer stem cell (CSC) model [1], which proposes that genomic alterations in tissue-specific cells results in clonal tumor cell populations with stem cell-like properties, including the capacity for self-renewal and differentiation [2]. Hence tumors following the CSC model comprise a cellular hierarchy of infrequent BTIC at their apex and an abundant non-tumorigenic cell population arising from BTIC at their base. Recent findings demonstrate that induction of an epithelial to mesenchymal transition (EMT) can endow non-tumorigenic breast tumor cells with BTIC activity implying that tumor cells transition between non-tumorigenic and tumorigenic states [3–5]. Hence, the abundant non-tumorigenic cell population may provide a reservoir of BTIC.

These observations have therapeutic implications [6–8]. Conventional cytotoxic therapies principally eradicate the abundant non-tumorigenic progeny of BTIC. Consequently tumors regress after cytotoxic therapies, but often recur likely due to therapy-resistant BTIC. Indeed the frequency of BTIC increases in tumors after neo-adjuvant chemotherapy [9] or after exposure of breast tumor cells to chemotherapy *ex vivo* [10, 11]. Consequently, to provide durable breast cancer remissions anticancer therapies should eradicate BTIC and their non-tumorigenic progeny.

Identifying molecular targets required to maintain BTIC activity would provide a means to develop anti-BTIC therapies. However, the latter has been difficult to achieve due to the scarcity of BTIC in human breast tumors [12] or breast tumor cell lines and the inability to sufficiently purify BTIC for molecular analyses [13]. We previously reported that tumors from multiple transgenic mouse models of breast cancer comprise a high BTIC frequency [14], which is maintained when the cells are propagated in chemically-defined, serum-free medium [15] as non-adherent spheres, which we termed tumorspheres [16]. The capacity to propagate BTIC-enriched tumor cells *in vitro* enabled a high-throughput phenotypic screen using a sensitive cell viability assay with approximately 35,000 compounds [17]. We found that neurotransmitter antagonists comprised a high frequency of the small molecules of known mechanism of action that affected the viability of sphere-derived mouse tumor cells. Moreover, we confirmed that the serotonergic antagonists we identified targeted mouse BTIC and the sphere-forming subpopulation of mouse tumorspheres. Herein we report that serotonergic pathway components are expressed in human breast tumor cell lines independent of the molecular subtypes they model, and that inhibitors of such proteins targeted BTIC and synergized with docetaxel (Taxotere) to shrink breast tumor xenografts.

## RESULTS

### *SLC6A4* expression and gene copy number variation in breast tumors

In advance of assessing the activity of 5-HT antagonists in human breast tumor cell lines we mined transcriptomic and genomic datasets of breast tumors to determine whether 5-HT signaling might be implicated in breast cancer. We focused primarily on SERT (encoded by *SLC6A4*) because its antagonists include SSRI, highly selective and safe drugs that are widely used to treat depression and other mood disorders.

**Figure 1:**
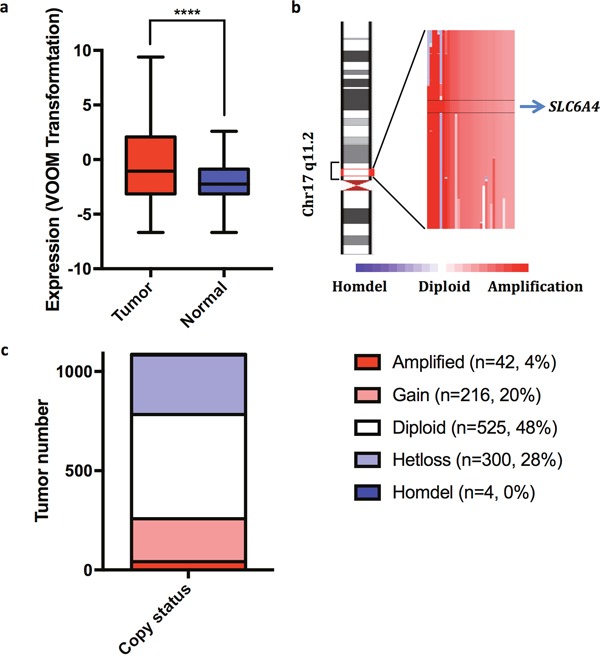
*SLC6A4* transcripts are overexpressed and the gene amplified in a fraction of human breast tumors **(a)** *SLC6A* transcripts are more abundant by 2.8 fold (*P* = 5.74 × 10^−5^) in human breast tumors (N = 1081) compared to normal breast samples (N = 111). **(b)** Illustration of the chromosomal region of *SLC6A4* that is amplified in a fraction of human breast tumors. **(c)** The copy number status of *SLC6A4* in breast tumors (N = 1,087).

We initially determined whether *SLC6A4* transcripts are differentially expressed in breast tumors compared to normal breast samples. We found that breast tumors overexpressed *SLC6A4* transcripts by an average of 2.8 fold compared to normal breast samples (Figure 1a). We also determined whether *SLC6A4* copy number varied among breast tumor samples and found that the gene is amplified in a fraction of human breast tumors (Figure 1b and 1c). These findings suggested a link between SERT and breast tumorigenesis.

### TPH1, 5-HT and SERT are expressed in breast tumor cells *in vitro* and *in vivo*

**Figure 2:**
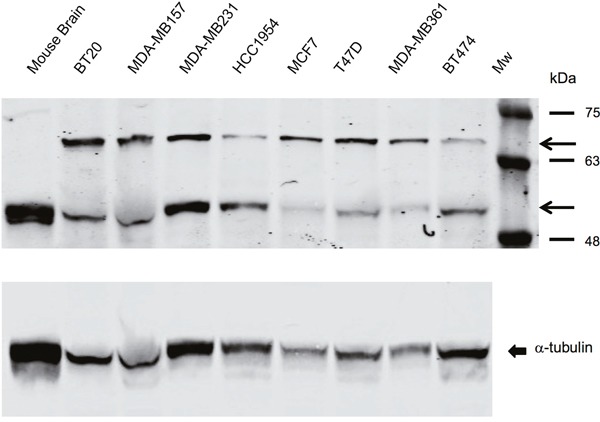
SERT is expressed in breast tumor cell lines representative of all the molecular subtypes of breast cancer Western blot performed with breast tumor cell line lysates (35 μg) and mouse brain tissue after electrophoresis in 5-15% gradient polyacrylamide gels reveal that the rabbit polyclonal antibody identifies SERT species that migrate between molecular mass markers of 63 kDa and 75 kDa, and between 48 kDa and 63 kDa. The arrowheads identify the glycosylated and non-glycosylated SERT species. Non-glycosylated SERT co-migrated with α-tubulin, the loading control, which is shown in the bottom-most panel.

Before testing the effect of serotonin antagonists in functional assays we determined whether SERT was expressed in breast tumor cell lines modeling the various molecular subtypes of breast cancer (Supplementary Table 1) [18]. We performed Western analyses of breast tumor cell line lysates with a polyclonal antibody obtained from rabbits immunized with a peptide comprising amino acids 388-400 of SERT, whose specificity has been previously validated [19]. Western analyses revealed that all the breast tumor cell lines expressed two SERT species identified previously at roughly the same level independent of the breast cancer molecular subtype that they mimicked (Figure 2) [19, 20]. The slowest migrating form of SERT detected with the polyclonal antibody represents the glycosylated form of the protein, whereas the faster migrating species is the non-glycosylated form of the protein, which co-migrated with SERT in mouse brain lysates and with the alpha (α)-tubulin loading control [19].

The natural substrate for SERT is 5-HT, which is synthesized from tryptophan. Tryptophan hydroxylase (TPH), the rate-limiting enzyme for 5-HT biosynthesis, converts tryptophan to 5-hydroxytryptophan (5-HTP), which in turn serves as a substrate for aromatic L-amino acid decarboxylase yielding 5-HT. There are 2 isoforms of TPH encoded by independent genes that are expressed in different tissues. TPH1 is expressed in a subset of cells of specific non-neuronal tissues such as enterochromaffin cells of the gastrointestinal tract, whereas TPH2 is expressed primarily in presynaptic neurons in midbrain raphe nuclei.

**Figure 3:**
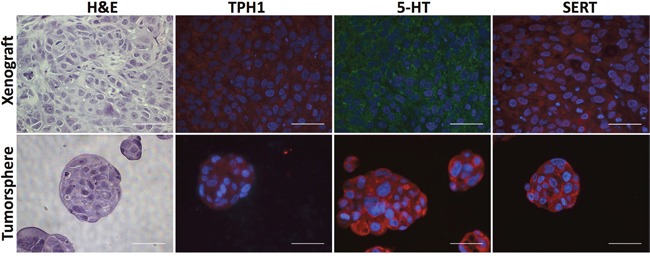
HCC1954 breast tumor xenografts and tumorspheres express TPH1, 5-HT and SERT Sections of xenografts or tumorspheres were stained with H&E to reveal their histology or incubated with antibodies specific to TPH1, 5-HT and SERT. Scale bars represent 50 micrometers (μm).

**Figure 4:**
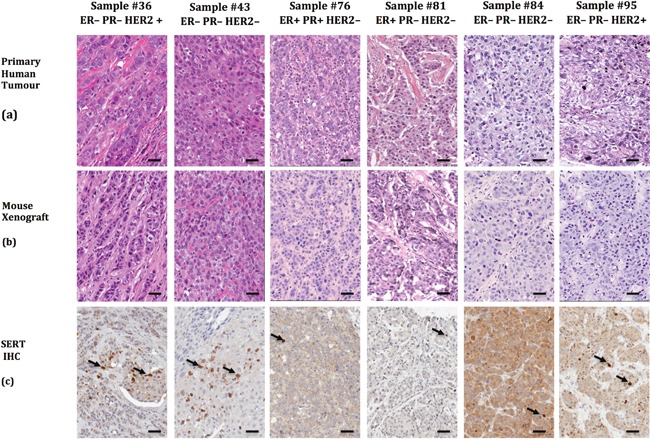
Patient-derived breast tumor xenografts recapitulate the phenotypic heterogeneity of the primary tumor from which they were derived and express SERT **(a)** Six primary human breast tumor samples and companion xenografts **(b)** were stained with H&E to reveal their histology. **(c)** SERT expression was detected by IHC in the PDX sections with the SERT-selective rabbit polyclonal antibody. The arrows identify tumor cells that express higher levels of SERT than the majority of the SERT-positive tumor cells in the same field. The scale bars represent 50 μm.

To learn whether TPH1 and 5-HT were expressed in breast tumor cell lines we used immunofluorescence (IF) staining with antibodies that bind to each of these molecules as well as to SERT. The majority of cells in the cell lines expressed TPH1, 5-HT and SERT (Supplementary Figure 1). TPH1, 5-HT and SERT were also expressed in most of the tumor cells in HCC1954 tumor xenografts and tumorspheres (Figure 3). We similarly detected SERT expression using immunohistochemistry (IHC) in sections of patient-derived breast tumor xenografts (PDX), which mimicked the histopathology of the primary tumors from which they were derived, and comprised each of the major clinical subtypes of breast cancer (Figure 4). Interestingly, the IHC analysis revealed variation in the intensity of SERT expression among the tumor cells in individual xenografts. Hence the machinery to synthesize and transport 5-HT is expressed in breast tumor cells regardless of their molecular or clinical subtype.

### Serotonergic pathway antagonists target sphere-forming cells resident in breast tumor cell lines

To determine whether serotonergic system antagonists altered tumor cell viability we determined their capacity to affect sphere formation, a functional assay for mammary epithelial stem cells (MESC) and BTIC [3, 21–25]. MESC [23] and BTIC co-fractionate with sphere-forming cells after fluorescence-activated cell sorting [13, 26], and agents that alter MESC/BTIC frequency similarly affect the frequency of sphere-forming cells [14, 27, 28] suggesting that MESC/BTIC possess sphere-forming activity.

We have shown that spheres arise from dispersed tumorsphere-derived cells in direct proportion to the number of cells plated into the medium, and that the frequency of sphere-forming cells, which averages 5% of the total tumor cell population in human breast tumor cell line-derived tumorspheres, can be accurately quantified over a range of cell densities [27]. Moreover plating single tumorsphere-derived cells into the wells of 96-well plates yields spheres at the same frequency as those forming when the cells are plated at higher cell densities [17].

We assayed the activity of 7 concentrations of compounds known to be selective for TPH1, SERT or each of 9 of the 14 human 5-HT receptors in HCC1954 breast tumor cells to establish their half maximal inhibitory concentration (IC_50_) in sphere-forming assays. Selective antagonists targeting TPH1, SERT and each of 7 5-HT receptors (5-HT_1B, 1D, 2A, 2B, 2C, 5A_, and _6_) inhibited sphere formation with an IC_50_ of less than 10 μM (Table 1).

**Table 1:** Selective antagonists of TPH1, SERT and 5-HT receptors inhibit tumorsphere formation by HCC1954 breast tumor cells

Target	Compound	IC_50_
TPH1	LP-533401	6.1
SERT	Paroxetine	2.7
	Fluoxetine	3.4
	Sertraline	1.1
5-HT_1B_	SB-224289	0.4
5-HT_1D_	GR-127935	4.8
5-HT_2A_	4F-4PP	2.2
5-HT_2B_	RS-127445	10.6
	SB-204741	8.2
5-HT_2C_	SB-242084	0.6
5-HT_4_	GR-113808	16.9
	SB-204070	22.3
5-HT_5A_	SB-699551	0.3
5-HT_6_	NPS ALX 4a	1.0
	SB-258585	9.1
5-HT_7_	SB-258719	14.0

Human HCC1954 cells were grown in the presence of serial dilutions of each selective antagonist. Over a four-day incubation many of the antagonists reduced sphere formation in a dose-dependent fashion. IC_50_ values (listed in μM) were calculated using Graphpad Prism 6. The activity of selective antagonists were determined in two biological experiments.

Because one of our goals is to provide pre-clinical data to determine whether existing FDA-approved drugs might be candidates for drug repurposing as anticancer agents we also tested a panel of serotonergic antidepressants for their capacity to affect sphere formation. All the antidepressants that were tested reduced sphere formation in a concentration-dependent fashion (Table 2).

**Table 2:** FDA-approved antidepressants inhibit sphere formation by HCC1954 breast tumor cells

Drug	Approved Drug Class	Molecular Target(s)	IC_50_
Clomipramine	Tricyclic Antidepressant	SERT, 5-HT_2A, 2C, 3, 6, 7_	1.9
Doxepin	Tricyclic Antidepressant	SERT, 5-HT_1A, 2A, 2C, 6_	4.1
Latrepirdine	Tricyclic Antihistamine	5-HT_2C, 5A, 6_	4.2
Cyproheptadine	Tricyclic Antihistamine	5-HT_1A, 2A, 2B, 2C, 3, 6, 7_	6.0
Mianserin	Tetracyclic Antidepressant	5-HT_1F, 2A, 2B, 2C, 6, 7_	12.5
Ziprasidone	Atypical Antipsychotic	SERT, 5-HT_1A, 1B, 1D, 2A, 2C, 6, 7_	0.8
Asenapine	Atypical Antipsychotic	5-HT_1A, 1B, 2A, 2B, 2C, 5A, 6, 7_	7.5
Vortioxetine	Selective Serotonin Reuptake Inhibitor	SERT, 5-HT_1A, 1B, 1D, 3A, 7_	1.1
Vilazodone	Selective Serotonin Reuptake Inhibitor	SERT, 5-HT_1A_	1.6
Fluoxetine	Selective Serotonin Reuptake Inhibitor	SERT, 5-HT_2A, 2C_	3.4
Paroxetine	Selective Serotonin Reuptake Inhibitor	SERT	2.7
Sertraline	Selective Serotonin Reuptake Inhibitor	SERT	1.1

HCC1954 tumorspheres were grown in the presence of different FDA-approved serotonergic antidepressants and their effect on sphere formation monitored. All the antidepressants reduced sphere formation in a dose-dependent fashion. IC_50_ values (listed in μM) were calculated using Graphpad Prism 6. The IC_50_ values of the drugs were determined in two biological experiments.

Our high-throughput screen, which identified serotonergic system antagonists as candidate anti-BTIC agents, was carried out in mammary tumor cells from an HER2 overexpressing mouse model of breast cancer [17]. Moreover, the HCC1954 breast tumor cell line we initially used for sphere-forming assays also overexpresses HER2 (Supplementary Table 1). Hence we sought to learn whether 5-HT antagonists affected the sphere-forming activity of breast tumor cell lines modeling different molecular subtypes of breast cancer by establishing their IC_50_ [18].

All the antagonists reduced the sphere-forming activity of the breast tumor cell lines (Table 3). The IC_50_ values of individual antagonists varied between some cell lines, but this difference was unrelated to their molecular subtype. Where differences in IC_50_ for a particular antagonist occurred between cell lines, these pair-wise comparisons invariably included the BT474 cell line. For example, the IC_50_ of 4F-4PP in the BT474 cell line (Luminal B) was greater than that in the MDA-MB-453 (Luminal B) or in the HCC1954 (Basal A) cell line by 14 fold and 4.5 fold respectively. Similarly the IC_50_ of SB-242084 in the BT474 cell line was nearly 8-fold higher than that in the HCC1954 cell line. These findings are likely related to the fact that BT474 tumor cells are chemoresistant due to the increased expression of ABC transporters [29].

**Table 3:** IC_50_ of selective 5-HT antagonists on sphere formation in a diversity of breast tumor cell lines

Target	Compound	1954	MCF7	ZR751	157	453	BT474	BT20	361	T47D	BT549
TPH1	LP533401	6.1	5.2	5.4	6.3	9.4	8.2	3.9		3.1	2.6
SERT	Sertraline	1.1	2.4	1.1	2.4	1.9	2.7	3.2	1.4	2.6	1.3
SERT	Vilazodone	1.6	2.7	3.0	2.5	1.4	2.2	2.1		2.2	1.7
5-HT_1B_	SB-224289	0.4	1.2	0.3	0.9	0.5	0.6				
5-HT_2A_	4F-4PP	2.2	3.7	6.4	10.6	0.7	10.0				
5-HT_2B_	RS-127445	10.6	5.3	11.7	12.2	4.8	16.2				
5-HT_2C_	SB-242084	0.6	1.6	2.8	2.7	1.8	4.6				
5-HT_5A_	SB-699551	0.3	0.2	0.2	0.3	0.1	0.3				
5-HT_6_	NPS ALX 4a	1.0	0.6	1.1	1.0	0.5	1.2				

Dispersed cells from each human breast tumor cell line were suspended in serum-free, chemically-defined medium and the effect of serial dilutions of the various compounds assayed for their capacity to affect sphere-formation. The compounds reduced sphere-formation in all cell lines in dose-dependent fashion. The IC_50_ (expressed in μM) of each compound in each cell line was calculated using GraphPad Prism 6. The IC_50_ values of the compounds in each cell line were determined in two biological experiments. The blank values illustrate examples of compounds that were not tested in select cell lines. The names of the HCC1954, MDA-MB-157, MDA-MB-361 and MDA-MB-453 cell lines were abbreviated 1954, 157, 361 and 453 respectively.

### SSRI target sphere-forming cells and BTIC by an irreversible mechanism

We previously reported that agents that irreversibly affect sphere formation induce cell death and differentiation programs, largely irreversible biological processes, which require a sustained period for execution and which reduce BTIC frequency [27, 30]. To learn whether SSRI acted by a reversible or irreversible mechanism to inhibit sphere formation, the spheres that arose 4 days after exposure of HCC1954 tumorsphere-derived cells to defined concentrations of either vilazodone or sertraline were dissociated, the same number of *viable* cells from each sample were seeded into SSRI-free medium for 4 days, and the number of spheres that arose in the secondary sphere-forming assays was determined and compared to those arising after exposure of the tumor cells to the vehicle.

**Figure 5:**
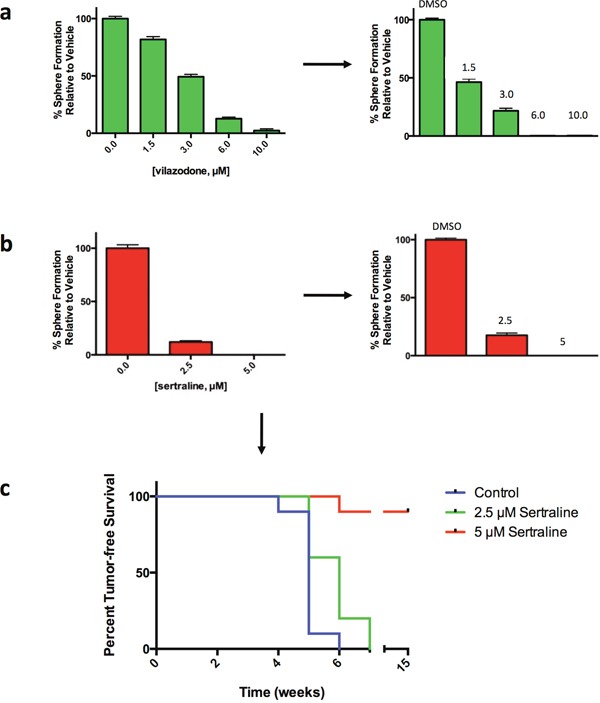
Vilazodone and sertraline target the sphere-forming tumor cell subpopulation and BTIC by an irreversible mechanism **(a)** Exposure of HCC1954 breast tumor cells to vilazodone in a primary sphere-forming assay (left-most panel) irreversibly reduced the frequency of sphere-forming cells in secondary sphere-forming assays performed in drug-free medium (right-most panel). **(b)** Exposure of HCC1954 tumor cells to sertraline in a primary sphere-forming assay irreversibly reduced the frequency of sphere-forming cells in secondary sphere-forming assays carried out in the absence of the drug. **(c)** Sertraline targets HCC1954 breast tumor-initiating cells. Dispersed HCC1954 cells from tumorspheres exposed to sertraline under sphere-forming conditions *ex vivo*, yield tumors after transplant into NOD/SCID mice with an increased latency and at a reduced frequency compared to tumor cells exposed to the vehicle. Mice were monitored for disease-free survival by Kaplan-Meier survival analysis and statistical significance was determined using a log-rank (Mantel-Cox) test: *P* = 3.0 × 10^−4^.

The tumorsphere-derived cells exposed to the vehicle formed spheres in the secondary sphere-forming assay at the same frequency (~5%) as they did in the primary sphere-forming assays (Figure 5a and 5b). By contrast, exposure of the tumorsphere-derived cells to each SSRI during the primary sphere-forming assays reduced the frequency of sphere-forming cells in a concentration-dependent fashion in the secondary sphere-forming assays. Hence both SSRI targeted the sphere-forming subpopulation of tumorspheres by an irreversible process.

The data showing that SSRI targeted sphere-forming cells raised the likelihood that they targeted BTIC. Tumor initiating cells, including BTIC, are functionally defined by their capacity to initiate tumors following transplant into rodents. Consequently we incubated dispersed HCC1954 tumorsphere-derived cells into medium with the vehicle or with sertraline at either 2.5μM (~IC_50_) or 5.0μM (~IC_90_). Following exposure of the cells to the vehicle or to sertraline, the spheres that formed were dissociated and an equal number of *viable* cells were transplanted into one of the #2 fat pads of 10 6-8 week old female non-obese diabetic/severe combined immunodeficiency (NOD/SCID) mice. We monitored tumor incidence during a 15-week period.

Tumor xenografts were detected in 1 of the 10 mice transplanted with vehicle-treated tumor cells 4 weeks following their transplantation, but by 6 weeks all the mice in this cohort had developed tumor xenografts (Figure 5c). Four of the 10 mice transplanted with tumor cells exposed to 2.5μM sertraline developed xenografts by 5 weeks post-transplant, but by 7 weeks all the mice had developed tumor xenografts. By contrast, only 1 mouse developed a xenograft 6 weeks after transplant of tumor cells exposed to 5.0μM sertraline, and all of the 9 remaining mice remained tumor free for up to 15 weeks, at which time the experiment was concluded. Hence exposure of tumor cells to sertraline reduced the incidence of tumor xenografts and delayed their appearance dependent on the concentration of sertraline to which they were exposed *ex vivo*. These findings are consistent with our previous observation [14] and those of others [31] that the incidence and time to appearance of tumors arising after transplanting tumor cells into mice is directly proportional to the frequency of BTIC in the transplanted tumor cell population.

### Vilazodone synergizes with docetaxel to shrink breast tumor xenografts

Any clinical studies to assess the efficacy of serotonergic drugs in breast cancer patients will likely be carried out by treating them with both a 5-HT antagonist and conventional breast cancer therapies. We also imagine that achieving durable breast cancer remissions in patients will require targeting both the BTIC tumor cell population and the non-tumorigenic tumor cell population, a potential source of BTIC. Hence we sought to determine whether vilazodone and docetaxel might be combined to affect tumor xenograft growth in mice.

**Figure 6:**
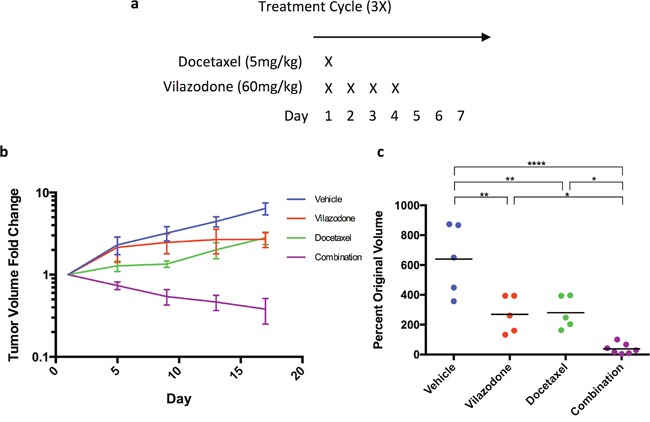
The combination of vilazodone and docetaxel synergistically shrink HCC1954 breast tumor xenografts **(a)** Docetaxel was administered on the first day of the treatment regimen, whereas vilazodone was administered on the first day of the treatment regimen and for 3 consecutive days thereafter. **(b)** Individually docetaxel and vilazodone reduced tumor growth rate as determined by changes in tumor volume with time, but together the drugs shrank tumors. **(c)** A week after the end of the treatment regimen the tumor xenograft volumes of mice administered vilazodone, docetaxel or the combination of both drugs were statistically significantly smaller than those administered the vehicle. One-way ANOVA *P* <0.0001.

We used vilazodone for these experiments because unlike some other SSRI vilazodone does not inhibit CYP2D6, which is required for the conversion of the breast cancer pro-drug tamoxifen into its active metabolite endoxifen, nor does it affect CYP3A4 activity, which metabolizes many chemotherapeutics [32]. We used HCC1954 tumor cells because they form xenografts more rapidly than any of the other breast tumor cell lines we tested (data not shown). In advance of performing the study we established the maximum tolerated dose of vilazodone in combination with docetaxel (5 mg/kg, half that which is normally employed) using the dosing regimen we planned to use in subsequent *in vivo* experiments (Figure 6a). The female NOD/SCID mice tolerated 60 mg/kg and lower amounts of vilazodone during a 3-week course of combination therapy, but animals administered higher doses became moribund or died (data not shown). Hence we used the 60 mg/kg dose for the preclinical study.

We orthotopically transplanted HCC1954 tumor cells dissociated from tumorspheres into NOD/SCID female mice to elicit xenograft growth. When the xenografts of mice averaged a volume of ~300 mm^3^ we treated the mice with the vehicle (5 mice), vilazodone (5 mice), docetaxel (5 mice) or a combination of vilazodone and docetaxel (7 mice) using the dosing schedule in Figure 6a. Treatment of mice with the drugs occurred over 3 weeks; the mice were sacrificed a week after the third treatment cycle. Tumor volume was measured every 4 days after the treatment started.

The tumor xenografts of the vehicle-treated mice increased in volume 6-7 fold during the time course of the experiment (Figure 6b). The xenografts of mice administered vilazodone or docetaxel also increased in volume after treatment started, but to a lesser extent (2-fold) than those of mice administered the vehicle. By contrast, the volume of the xenografts of mice treated with both vilazodone and docetaxel decreased after the first treatment cycle and continued to decline after the third treatment cycle (Figure 6c). Four of the xenografts remaining after combination therapy were nodules and 2 others were blood filled (Supplementary Figure 2). Hence the combination of both drugs reduced xenograft growth to a much greater extent than did each drug individually suggesting that the drugs functioned synergistically. These findings recapitulate our previous observations where we showed that the combination of docetaxel and sertraline functioned synergistically both *in vitro* and *in vivo* using mouse mammary tumor cells [33]. Our findings are consistent with the hypothesis that each drug targets a different tumor cell population; docetaxel likely eliminates the non-tumorigenic tumor cells whereas vilazodone or sertraline eradicates the BTIC, which serve as the source of the non-tumorigenic tumor cells.

**Figure 7:**
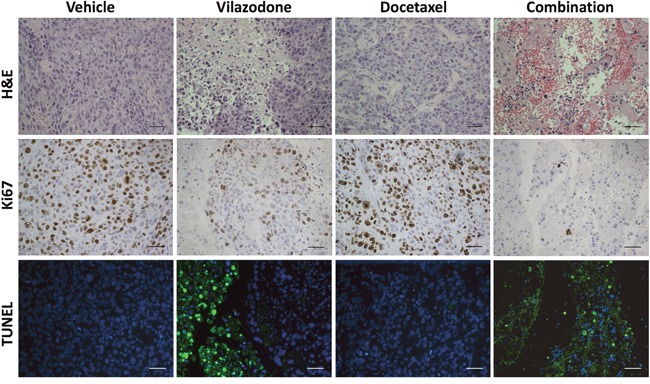
The combination of vilazodone and docetaxel reduces the frequency of proliferating tumor cells and increases that of apoptotic cells in tumor xenografts to a greater extent than do either compound individually Xenografts resected from mice that were administered the vehicle, vilazodone, docetaxel or a combination of each drug were fixed, embedded, sectioned and stained with H&E, antibodies to Ki67 or assayed for fragmented DNA using the TUNEL assay. The various panels from each xenograft were prepared from consecutive sections. The scale bars represent 50 μm.

To uncover potential mechanisms by which vilazodone and/or docetaxel limited xenograft growth, we prepared sections from the tumor xenografts at the end of the treatment period and stained them with hematoxylin and eosin (H&E) to reveal their histology. The xenografts of mice treated with vilazodone were morphologically distinct from those of mice administered the vehicle comprising clusters of tumor cells separated by cell-free areas, which contained stromal cells and extra-cellular debris (Figure 7). Xenografts from docetaxel-treated mice appeared histologically similar to those of the vehicle-treated mice. By contrast, the xenografts harvested from mice that were treated with the drug combination were largely devoid of tumor cells, and comprised red blood cells as well as regions of stromal cells and cellular debris.

To learn what cellular processes were affected by vilazodone, docetaxel or their combination we enquired whether their effect on tumor growth could be ascribed to changes in tumor cell proliferation or apoptosis. We used immunohistochemistry (IHC) with antibodies to Ki67 to estimate the frequency of proliferating cells in tumor sections from all 4 treatment-groups at the end of the treatment period. Neither vilazodone nor docetaxel individually affected the frequency of Ki67-positive tumor cells (Supplementary Figure 3). However, we observed a substantial decrease in Ki67-positive cells in xenograft sections from mice treated with the combination of vilazodone and docetaxel compared to those of mice administered the vehicle or each drug individually.

TUNEL assays with xenograft sections demonstrated that vilazodone increased the frequency of apoptotic cells, whereas docetaxel had little effect (Figure 7). Xenografts of mice administered both drugs comprised fewer tumor cells, which occurred in clusters, and any remaining tumor cells in surrounding regions were undergoing apoptosis. These observations suggest that the drug combination shrank the xenografts by inhibiting tumor cell proliferation and by inducing their apoptosis. Kinetic analyses of tumor cell proliferation and apoptosis during the treatment period would provide stronger support for the conclusion that the reduced rate of tumor xenograft growth results from changes in these cellular processes.

## DISCUSSION

Our data demonstrate that breast tumor cells possess the enzymatic machinery to synthesize 5-HT, which acts by an autocrine or paracrine mechanism in conjunction with other serotonergic pathway components to maintain BTIC activity. Structurally unrelated selective antagonists affecting the activity of TPH1, SERT and a minimum of 7 individual 5-HT receptors reduced the frequency of BTIC as revealed by sphere-forming or tumor cell transplantation assays. The fact that inhibitors of TPH1, SERT and any one of several receptors compromised BTIC activity suggests that each protein plays a distinct role in BTIC biology. Whereas the role of TPH1 seems obvious that of SERT and those of several independent receptors is not yet clear. The observation that the combination of vilazodone and docetaxel synergized to shrink breast tumor xenografts is consistent with the hypothesis that each drug targets a different but interrelated tumor cell population, likely BTIC and their non-tumorigenic descendants.

Genetic evidence demonstrating a requirement for 5-HT function to maintain BTIC activity would secure the role of the serotonergic system in breast cancer, and provide insight into the biological roles of its individual components. Interestingly knockout mice homozygous for genes encoding several of the serotonergic system proteins, including *SLC6A4*, have been isolated demonstrating that individually these genes are not essential for embryonic development or adult tissue physiology and homeostasis [34–36].

Hence, we attempted to knockout *SLC6A4* in breast tumor cell lines using the CRISPR-Cas9 genome-editing tool with the expectation that cells lacking the gene might be viable. Whereas we were able to successfully target 1 of the 2 *SLC6A4* alleles in diploid MCF-7 cells, and 3 of the 4 alleles in tetraploid HCC1954 tumor cells we were unable to derive a single clone from either cell line lacking functional SERT among hundreds of clones that were screened, implying that the gene is essential for the viability of these breast tumor cell lines (data not shown). In this regard it is noteworthy that knockout of *Slc6a14*, which encodes a transporter for neutral amino acids including tryptophan, the precursor of 5-HT, compromises mammary tumorigenesis in a mouse model of breast cancer [37]. Our current efforts are centered on conditionally targeting *SLC6A4* and the genes encoding *TPH1* and each of the 14 5-HT receptors in human breast tumor cell lines to learn whether loss of each gene phenocopies the effect of selective serotonergic antagonists of their encoded molecular targets.

To learn whether previous functional genomic screens identified genes encoding serotonergic pathway components as being essential for the proliferation of human tumor cell lines *in vitro*, we mined the data resulting from genome-wide CRISPR-Cas9 knockout screens in human cancer cell lines [38, 39]. The cell lines included 2 chronic myelogenous leukemia cell lines and 2 Burtkitt's lymphoma cell lines in one study [38], and a colon carcinoma, cervical carcinoma, glioblastoma and melanoma cell line in another [39]. All the cell lines were propagated in serum-containing medium as adherent cultures and passaged at least 3 times. Under the conditions of the latter studies none of the genes encoding serotonergic pathway proteins were identified as essential for tumor cell proliferation. However, none of the cell lines investigated were of breast origin. Hence we mined the data resulting from a recently reported shRNA dropout screen performed in over 70 breast tumor cell lines that were propagated as adherent cultures in serum-containing medium for 2 passages [40]. shRNAs targeting transcripts encoding TPH1, SERT and many of the 5-HT receptors statistically significantly dropped out during the propagation of the breast tumor cell lines *in vitro* (data not shown). This latter study is in accord with our unpublished data suggesting that *SLC6A4* is essential for the viability or proliferation of the MCF-7 and HCC1954 breast tumor cell lines.

IF and IHC analyses of 5-HT pathway component expression in breast tumor cell lines revealed their presence in a fraction of cells that supersedes the frequency of BTIC reported in these sources [1, 13, 41]. One explanation for this discrepancy is that the extent to which the 5-HT pathway proteins are expressed differs between BTIC and their non-tumorigenic progeny. The latter is suggested by our IHC analyses of PDX, which revealed that infrequent SERT-positive cells in PDX were more intensely stained than others. Additional experiments are required to determine whether both the tumorigenic and non-tumorigenic tumor cells in PDX and other sources differentially express the various 5-HT pathway proteins, and whether their expression correlates with BTIC activity.

A link between 5-HT and breast cancer has been reported in previous studies. Analysis of 288 breast tumors revealed increased expression of TPH1 in tumors compared to normal breast samples, and an association between increased TPH1 levels and breast cancer progression [42]. Another study uncovered a correlation between increased plasma-free 5-HT and the risk of breast cancer recurrence in a cohort of 29 women [43]. Moreover, recent analyses of transcriptomic and metabolomic data from thousands of breast tumor specimens demonstrated a correspondence between patients predicted to have a poor prognosis and increased tumor-specific 5-HT production [44]. Breast tumor recurrence and poor patient prognosis may be attributable to BTIC, which require 5-HT for their activity, seed metastases and are refractory to conventional therapies [45, 46].

Studies pioneered by Horseman and his colleagues were the first to implicate 5-HT in postnatal mouse mammary gland development [47, 48]. Initial studies identified *Tph1* as a prolactin target gene [47]. TPH1 transcripts are increased during pregnancy and lactation leading to increased 5-HT levels in the mammary epithelium. 5-HT acts in a negative feedback loop to suppress prolactin stimulation of milk production during lactation and to initiate involution by inducing epithelial cell apoptosis [48]. The function of 5-HT requires 5-HT receptor activity because a non-selective receptor antagonist, methysergide, inhibited the effect of 5-HT on the expression of milk proteins and 5-HT mediated initiation of apoptosis [47]. 5-HT also increased the synthesis of parathyroid hormone-related peptide (PTHrP), which acts on bone to release calcium that ultimately accumulates in milk. Recent publications demonstrate that 5-HT binding to the 5-HT_7_ receptors triggers mammary epithelial cell apoptosis during the involution phase of postnatal mammary gland development, whereas 5-HT binding to the 5-HT_2B_ receptor stimulates the expression of PTHrP from mammary epithelial cells during lactation [49–53]. Hence 5-HT binding to different receptors in the mammary gland regulates different cellular processes likely by activating distinct signaling pathways. By analogy, our findings of a requirement for multiple 5-HT receptors for BTIC activity in sphere-forming assays may reflect the fact that each receptor acts through a distinct signaling pathway, and that activation of multiple pathways is required to maintain BTIC activity.

It is noteworthy that the serotonergic system has been implicated in other malignancies including lymphoma and leukemia, prostate carcinomas, small cell lung carcinomas, glioblastomas, bladder carcinomas, colorectal carcinomas, hepatocellular carcinomas, cholangiocarcinomas, choriocarcinomas, carcinoid tumors and ovarian tumors (reviewed in [54]). Interestingly, overexpressing cDNAs encoding the 5-HT_2C_ or 5-HT_2A_ receptors results in the transformation of mouse 3T3 fibroblasts demonstrating that they function as oncogenes in the focus-forming assay [55, 56]. Consistent with our findings serotonergic system antagonists, including several that we independently discovered [17], were recently found to inhibit sphere formation by glioblastoma cell lines [57] and to affect the growth of tumor allografts and xenografts of neuroendocrine origin [58].

Epidemiologic studies have sought to determine whether SSRIs increase breast cancer recurrence, as a consequence of findings in experimental rodent models in the early 1990s suggesting that SSRI increased the incidence of tumors (reviewed in [59]). However, subsequent studies found that there is no association between SSRI use and breast cancer risk in women. To the best of our knowledge epidemiological studies have not addressed whether antidepressants reduce the risk of breast cancer, or whether their use during cytotoxic anticancer therapies reduces breast cancer recurrence.

The repurposing of serotonergic antagonists to treat breast cancer will depend in part on whether therapeutic concentrations can be achieved in patients. Two of the SSRI we used here (sertraline and vilazodone) have IC_50_ values *in vitro* between 1- 2 μM in a diversity of human breast tumor cell lines. The concentration of sertraline in the plasma of individuals who were orally administered 200 mg of the drug is 0.19 ug/ml (0.55 μM), which was achieved between 4.5 - 8.4 hours post administration: the half-life of the drug is between 24 and 36 hours [60]. The plasma concentration of sertraline is directly proportional to the administered oral dose over the range of 20 - 400 mg. Moreover, daily oral doses of 400 mg of sertraline are well tolerated suggesting that a 1μM plasma concentration, the approximate IC_50_ of the drug in breast tumor cell lines, can be achieved in humans [61]. Vilazodone can similarly achieve plasma concentrations in humans at the IC_50_ required to inhibit sphere formation *in vitro* [62]. The fact that the SSRI we have tested synergize with docetaxel when used at their respective IC_50_ values in human breast tumor cell lines offers the promise that therapeutic doses of these SSRI can be achieved in breast cancer patients.

Collectively our data imply that 5-HT signaling is required to maintain BTIC activity and suggests that drugs affecting the serotonergic system might be repurposed to treat breast cancer patients in combination with anticancer therapies to achieve more durable breast cancer remissions than occur currently. SSRI in particular are among the most widely prescribed antidepressants, have been used for decades and are considered safe when used as prescribed suggesting that their testing in clinical trials as anticancer drugs may be warranted [63].

## MATERIALS AND METHODS

### Care and treatment of mice

All procedures involving mice were performed with the approval of the Canadian Council on Animal Care.

### Patient-derived xenografts

The Hamilton Integrated Research Ethics Board approved all protocols associated with the collection of primary human breast tumor samples. Primary patient breast tumors were processed to yield dispersed cells and these transplanted into the cleared humanized #4 mammary gland of NOD/SCID gamma mice (NSG) [64]. Tumor xenografts were subsequently propagated in NOD/SCID mice [14].

### Analyses of *SLC6A4* transcripts and copy number

All genomic data was publicly available. Level 3 DNA copy number data for breast tumors were downloaded from the BROAD Institute portal (https://gdac.broadinstitute.org/) on June 28, 2016 and level 3 RNA sequencing data (RSEM) for breast tumors and normal tissues were obtained from the National Cancer Institute GDC Data Portal (https://gdc-portal.nci.nih.gov/) on August 22, 2016. GISTIC 2.0, which facilitates sensitive and confident localization of focal somatic copy number alterations, was used to estimate the copy number of *SLC6A4* [65]. Sequencing data were normalized with the TMM normalization method [66] and then transformed with voom transformation [67]. Thereafter, differential expression analysis was performed using the *limma* package in R [68].

### Cell culture

Breast tumor cell lines were purchased from the ATCC (Manassas, VA, USA) and grown in serum-containing medium with the recommended supplements. Breast tumor cell line derived tumorspheres were treated with trypsin (0.25%) to yield dispersed cells, which were placed in chemically-defined, serum-free medium containing B-27, Epidermal Growth Factor, Fibroblast Growth Factor 2 and heparin to form tumorspheres [14]. The tumorspheres were passaged every 4 days by trituration and exposure to trypsin followed by reseeding the dispersed cells into fresh medium.

### Sphere-forming assays

Quantitative sphere-forming assays were performed as described [17, 27].

### IC_50_ calculations

The IC_50_ of compounds was calculated using GraphPad Prism 6 software as described [27, 30]. To aid IC_50_ calculations, the vehicle comprised a 1 nanomolar concentration of the tested compound.

### Animal studies

The effect of vilazodone and docetaxel on HCC1954 breast tumor xenograft growth was performed as described [30]. Drug treatments were initiated when the average xenograft volume was ~300 mm^3^. The mice were randomly assigned to each of the treatment cohorts. Tumor volume was measured every 4 days after treatment started.

### Histology and protein analyses

Tumor xenografts were processed to obtain sections, and IF and IHC analyses of the sections were performed as described [14, 27, 30, 69, 70]. The proteins in tumor cell lysates were resolved by electrophoresis in denaturing conditions in 5-15% gradient polyacrylamide gels and immunoblots were carried out as described [71]. To identify human SERT in tumor cell lysates we used a polyclonal antibody to SERT (Alomone Labs; Jerusalem, Israel), elicited by immunization of rabbits with a peptide corresponding to amino acids 388-400. The polyclonal antibody was also used to detect SERT in breast tumor xenograft sections as described [17]. A polyclonal antibody to TPH1 (LifeSpan BioSciences, Inc.; Burlington, ON, Canada) generated by immunizing rabbits with a peptide (amino acids 231-280) was used to detect the protein in breast tumor cell lines [17]. A mouse monoclonal antibody to 5-HT (Novus Biologicals; Oakville, ON, Canada) was used to detect the neurotransmitter in breast tumor cell lines [17]. Western blots IF and IHC with primary and secondary antibodies were performed in accordance with the recommendations of their manufacturers or as we described previously [17]. Color images were converted to grayscale, inverted, and their contrast adjusted to optimal resolution using ImageJ software. An antibody to Ki67 (ABCAM, Cambridge, Mass., USA) was used to identify proliferating tumor cells, whereas TUNEL assays were performed to identify apoptotic cells in xenograft sections as described [27, 30].

### Statistical analyses

Assays were repeated in 2 or more biological experiments with each data point being the average of a minimum of 3 technical replicates. Where relevant the figures show the mean +/− the standard error. Differences among experimental means were analyzed by analysis of variance (one-way ANOVA) using Graphpad Prism 6 (La Jolla, CA., USA). Significant differences between individual means were calculated using Tukey's test. For Kaplan-Meier survival, significance was determined using a log-rank (Mantel-Cox) test. Differences were considered statistically significant if *P* < 0.05.

## SUPPLEMENTARY MATERIALS FIGURES AND TABLES


