# Supplementary material for: Serotonergic system antagonists target breast tumor initiating cells and synergize with chemotherapy to shrink human breast tumor xenografts
Source: Oncotarget. 2017 Mar 29;8(19):32101–16. doi: 10.18632/oncotarget.16646 (PMC5458271; doi:10.18632/oncotarget.16646)
Supplement: Supplementary file 1 [file oncotarget-08-32101-s001.pdf]

Serotonergic system antagonists target breast tumor initiating cells and synergize with chemotherapy to shrink human breast tumor xenografts

SUPPLEMENTARY FIGURES AND TABLE

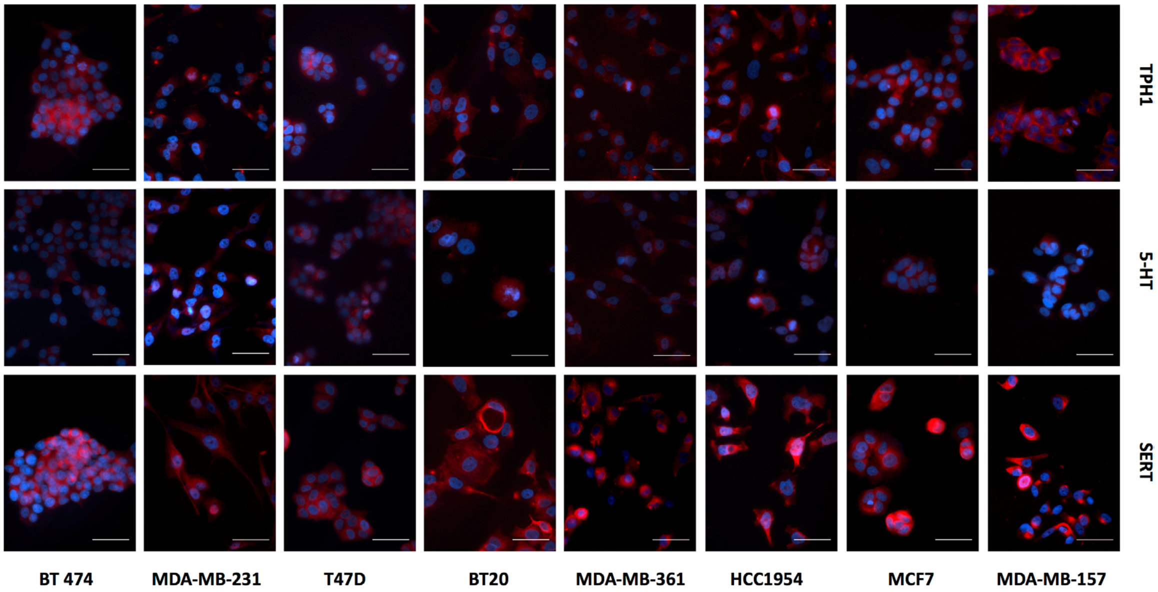

Supplementary Figure 1: Human breast tumor cell lines synthesize 5-HT, and express both TPH1 and SERT. Cells grown as adherent cultures in serum-containing medium were fixed and incubated with antibodies that specifically bind to TPH1, 5-HT and SERT. Immunofluorescence imaging reveals expression of TPH1, 5-HT and SERT in the majority of the cells of each breast tumor cell line.

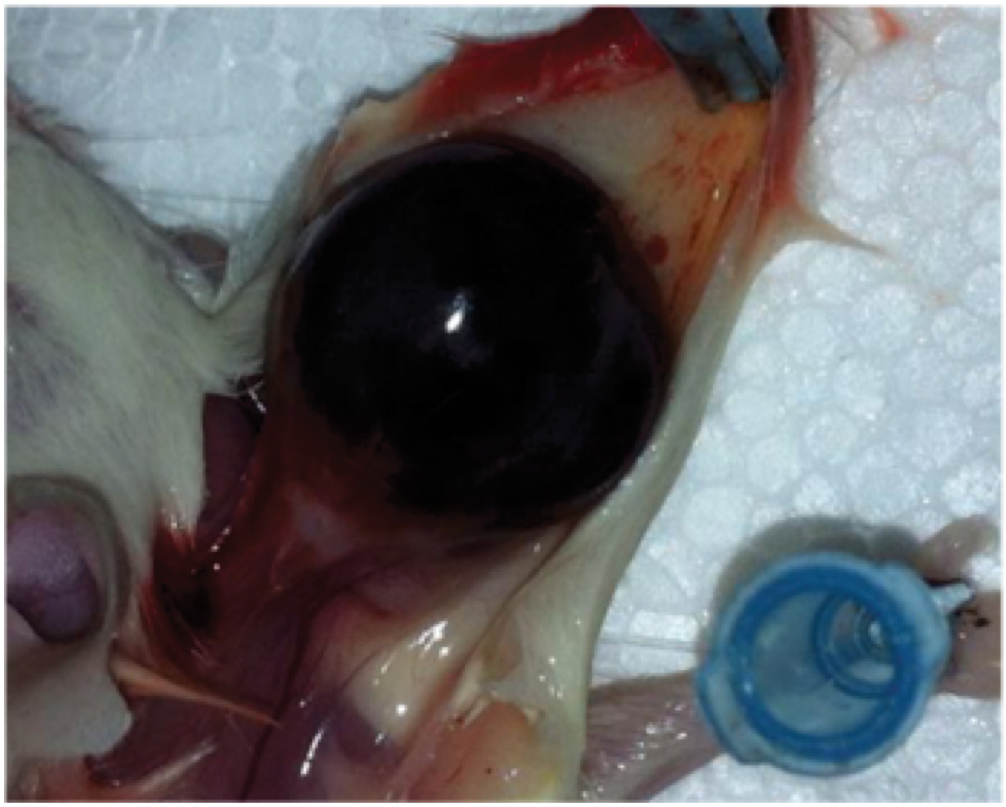

Supplementary Figure 2: A blood-filled residual tumor xenograft remaining after exposure of mice to the combination of vilazodone and docetaxel.

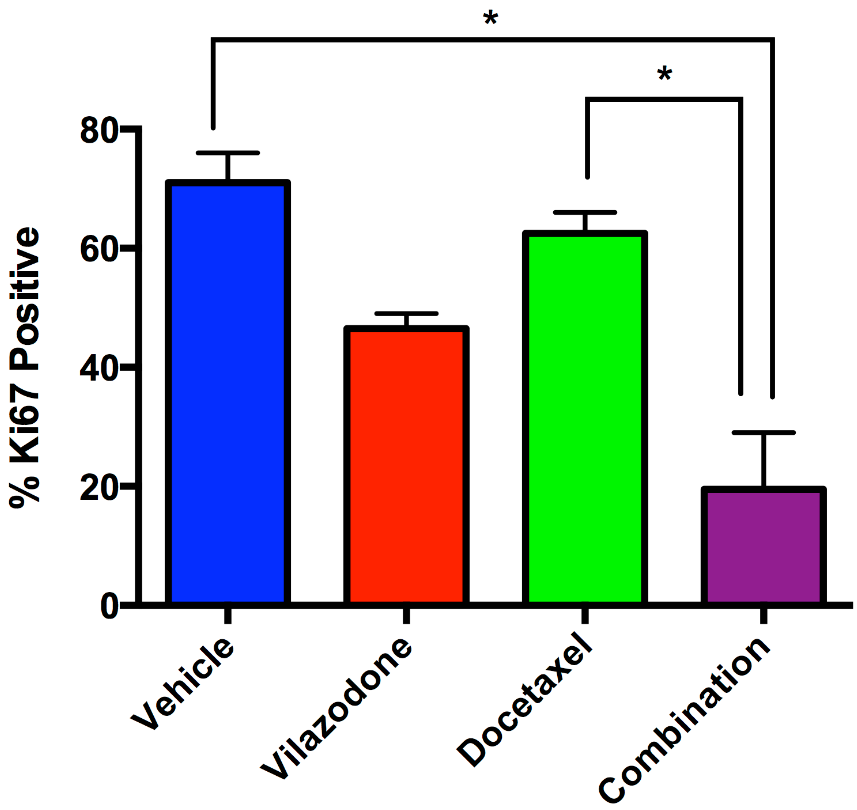

Supplementary Figure 3: Vilazodone in combination with docetaxel reduced the frequency of proliferating cells to a greater extent than did either compound individually. Sections of residual tumor xenografts remaining after drug treatment were stained with antibodies to Ki67. The fraction of Ki67-positive cells was calculated in two independent tumor xenografts from each treatment cohort and expressed as a percentage of all the tumor cells. *P*-values ($P = 0.01$) were calculated using one-way ANOVA.

Supplementary Table 1: Subtype of human breast tumor cell lines

Cell Line	Molecular Subtype	ER	PR	HER2
HCC1954	Basal A	-	-	+
MCF7	Luminal A	+	+	
ZR751	Luminal A	+	+	
MDA MB 157	Basal B	-	-	
MDA MB 453	Luminal B	-	-	-
BT474	Luminal B	+	+	+
BT20	Basal A	-	-	
MDA MB 361	Luminal A	+	+	+
T47D	Luminal A	+	+	
BT549	Basal B	-	-	

The molecular and clinical subtype (estrogen receptor [ER], progesterone receptor [PR], and HER2 status) of human breast tumor cell lines.
